# Supplementary figures and images for: RNAseq Analysis of Novel 1,3,4-Oxadiazole Chalcogen Analogues Reveals Anti-Tubulin Properties on Cancer Cell Lines
Source: Int J Mol Sci. 2023 Jul 9;24(14):11263. doi: 10.3390/ijms241411263 (PMC10379353; doi:10.3390/ijms241411263)

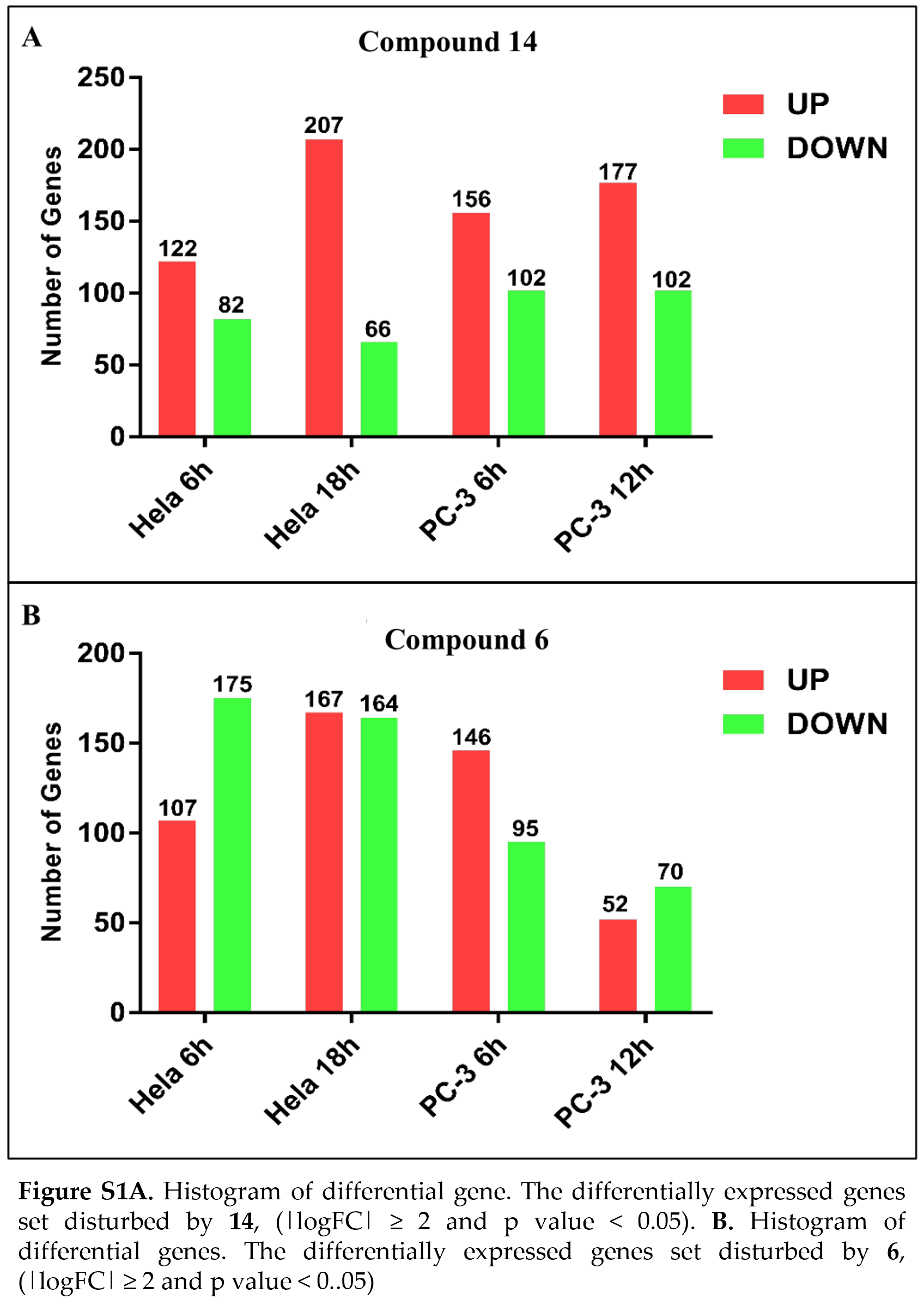

Supplement: Supplementary file 1 [file ijms-24-11263-s001.zip › Figure S1A-1B.tif]

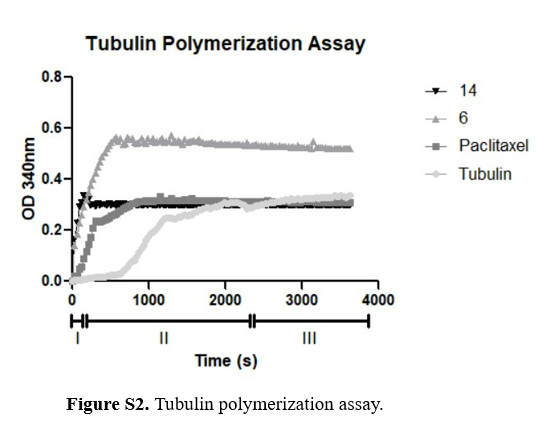

Supplement: Supplementary file 1 [file ijms-24-11263-s001.zip › Figure S2.tif]

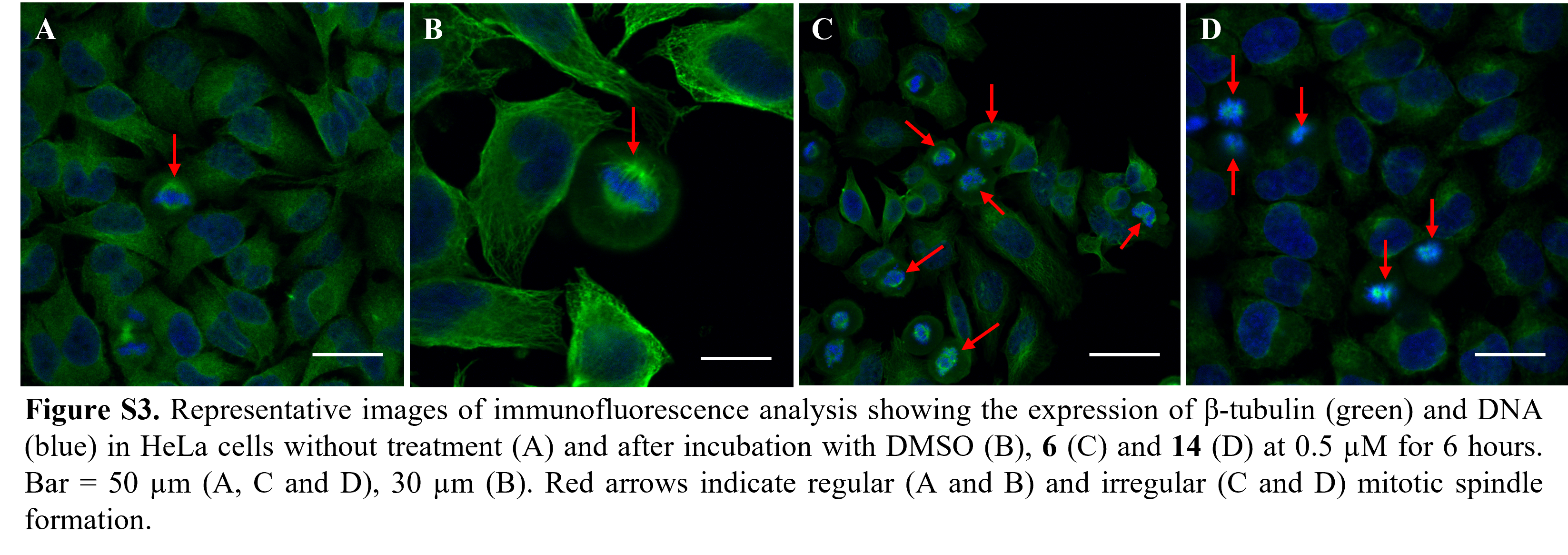

Supplement: Supplementary file 1 [file ijms-24-11263-s001.zip › Figure S3.tif]
